# The Hot Springs of Arkansas

**Published:** 1878-03

**Authors:** J. L. White


### THE HOT SPRINGS OF ARKANSAS.

*To the Editor of the Medical Journal and Examiner:*

It has become quite fashionable in this vicinity, for persons suffering from chronic ailments, of whatever nature, to visit the hot springs of Arkansas. Many having received benefit, the fashion is likely to be perpetuated and the profession often consulted with reference to such visits. Thinking that the results of personal experience may furnish some data for advice of this sort, I subjoin the observations made by myself in the short space of three weeks.

The hot springs of Arkansas are quite numerous—some 50 or more—all issuing from the base of Hot Springs Mountain, and confined to a comparatively small area. I should think the two most distant were less than seventy-five rods apart. The analysis of the waters of the different springs gives nearly the same results, with one exception. All the springs have, in my opinion, a common source, the difference being due to the different character of the material through which the water flows. The one spring materially differing from the others, evidently passes through a bed of magnesia, where, uniting with the carbonic acid, with which the water from all the springs is highly charged, is formed a deposit upon the mountain's side of almost pure carbonate; with this exception, the waters are nearly free from foreign matters of every kind. Numerous analyses have been made, all showing a large amount of carbonic acid, and but little else, the water averaging only about one grain of solid matter to the pint. It seems to me that somewhere in the laboratory of nature, this water has been condensed, and that the rock through which it subsequently passed, was so hard as to yield but very little of its constituent particles. And we find that most of the rock in the vicinity is flinty in its character, indeed, much of it is pure hornblende. Through such rock we can readily imagine distilled water might pass for a long distance without losing its purity. The general appearance of the country, the position of the rock, and indeed, everything clearly indicate excessive volcanic action; and my theory is, that perhaps at some distance, this action is still going on, and that steam is continually being driven into a cavern, where condensation takes place, whence the water, still heated, is driven by various courses, till at last it reaches the surface, somewhat singularly, in so nearly the same locality. We find these various springs so close together, differing in temperature very materially, the range being from about 86 to 160° Fahrenheit. The water thus heated, and thus pure, and thus flowing from the springs to the amount of upwards of 500,000 gallons per day, is used for drinking and bathing with general results, which I will describe as nearly as possible. Individual idiosyncrasies and pathological conditions change results very considerably, and render the study of each individual case necessary, as will readily be perceived when I come to describe the specific action—if it may be so called—of the water. Most persons prefer to drink the water as hot as they well can, say at about 140°, though at any temperature it is pleasant, and does not produce the slightest nausea. This quality is, I presume, mainly attributable to the carbonic acid gas it contains, as is also the sense of exhilaration which follows the draught, and which suggests the effect produced by drinking a cup of green tea. It requires but a short time to establish a liking for this beverage, and to feel a sense of discomfort when it cannot be obtained. The hotter it is taken, the more stimulating it seems to be, and the same is true to a greater extent of the bath.

In the bath houses appliances for every conceivable form of bath are to be found, but for my present purpose it is sufficient to speak of the simple tub bath. On entering the bath one is surprised at the buoyancy of the water. It is more buoyant than ordinary salt water, and yet nearly perfectly free from organic matter. Remaining six or eight moments in the bath, and at the same time sipping the hot water, a sensation of exaltation or stimulation is experienced, and an examination of the pulse reveals the fact that it has increased in frequency from five to ten beats per minute, provided the temperature of the bath was not above 98°. If above this degree of heat, these symptoms will be aggravated and a disagreeable sense of cerebral congestion, with its attendant symptoms, will exist. On emerging from the bath, after having been thoroughly rubbed, one is surprised at the prominence and fullness of the veins. A very casual observation will convince any one that in a very few moments there has been a material increase in the amount of the circulating fluid. The water is in such a condition that it enters with extreme readiness by endosmosis into the vessels, both from the stomach and through the skin; and, on purely mechanical principles, the volume being increased within given bounds, there must be a corresponding increase in the velocity. I saw one case in which, after a bath of eight minutes in a temperature of 104°, the pulse increased from 80 to 120 beats per minute, and I produced a similar, though not quite so great a rise, by experimenting upon myself. Relief from this condition comes from profuse perspiration or diuresis, one or both of which are sure to follow. The skin, aroused to greatly increased activity, becomes soft, smooth and pliable, and the process daily repeated all the tissues become washed out, as it were, and nature is thus aided in her efforts to remove not only the products of physiological disassimilation, but also any toxic agents which perchance may have found their way into the system. In this way also the removal of deposits and indurations is favored; but as every physician will readily perceive there is danger, if the vessels are weak, of a rupture and consequent hemorrhage, and there is also danger if any organ of the body is very much congested or diseased, of so overcrowding the vessels in it as to produce a stasis of the blood, just as a panic stricken crowd in a building blocks up the place of egress and prevents any one from escaping.

It thus will become very apparent to all that these waters are a powerful agent for evil as well as good unless judiciously employed. As an evidence of the correctness of the view advanced, I will state that a number of cases of sudden death were reported to me by physicians and keepers of bath houses, occuring either while the patient was in the bath or shortly after being removed. The proprietor of one of the houses reported to me the case of a man who insisted upon having his bath hotter than directed and remaining in for a longer time. On coming out he fell down in a semi-unconscious condition, and very soon commenced to vomit and purge exactly as if affected with cholera. The superabundance of serum was poured out into the stomach and bowels, and his life probably saved thereby. It is a matter of daily occurrence to hear persons say they have a violent headache in consequence of having taken their bath too hot that morning. I was credibly informed by physicians that it was not at all uncommon for ladies who had ceased to menstruate for a year or two, and who supposed the climacteric period passed, to have menstruation re-established for a time after taking a few baths, and Dr. Garnet informed me of a case in which it returned after an absence of eight years. It will thus be seen that no person inclined to the hemorrhagic diathesis, should be advised to visit these springs, nor should any one having cardiac disease, or very serious organic disease of the brain, or any of the visceral organs involving tissue destruction, or any great amount of consolidation. Where inflammation or congestion is not excessive, relief is sometimes afforded, I presume upon the same principle that a poultice will sometimes favor the resolution of a local inflammation by producing relaxation and increasing the circulation through the part.

In the treatment of the various forms of syphilitic disease, the springs have acquired their greatest reputation, but to the syphilitic virus, the waters furnish nothing antidotal; they are mainly aids to treatment. I heard of cases among the poor that were getting well under the use of the waters alone; but I took especial pains to visit all I could hear of, and I found every one making any manifest improvement managed in some way to get mercury and iodide of potash. The testimony of all the physicians is to the effect that secondary and tertiary syphilis is at the springs effectually and permanently cured in time, as it cannot be elsewhere, and such I am inclined to believe is the fact. All cutaneous diseases, and what are known as blood poisons, are benefited. Under the last head, I suppose rheumatism may be classed. In cases of nervous prostration and paralysis, due to anæmia, the use of the waters, together with a judicious general tonic course of treatment, produces very happy results in many instances. There are, however, in my judgment, other reasons to which these results may be attributed, in part, at least. It has never fallen to my lot to see a place where so much bragging is done as here. All are made to believe they will surely get well, and cases of miraculous cures in ailments similar to theirs are constantly being rehearsed to every invalid. A spirit of cheerfulness pervades the place, and sweet breathings of hope inspire every heart. The effect of the hot bath is to soothe and quiet nervous irritation, and the stimulating quality increases the appetite and aids digestion. Nearly all who take the baths eat heartily and sleep well.

The physicians of the place claim unusual success in the treatment of non-malignant uterine affections, which success they do not claim as the result of any extraordinary skill on their part, but as in the case of syphilis, to the baths as an adjuvant to treatment. One thing is certain, a large number of cases of this kind go there for treatment and most generally derive benefit. There are, however, some objections, as yet, to the place for this class of patients. Hot Springs is and probably always will be the Mecca for unfortunates afflicted with syphilis, and the idea of patronizing a bath-house, common and open to all kinds of cases, cannot be very pleasant to ladies of refinement. One among the most prominent physicians of the place has it in contemplation to erect an infirmary, with appropriate baths, for the reception of these cases, and to make a specialty of their treatment, moving with his family into the building, and making a home for those ladies who may choose to place themselves under his treatment. Something of this kind is needed, and should receive the encouragement of the profession. I would here remark it almost seems as if the waters promised some disinfectant property. In the holes on the mountain's side, where those too poor to patronize bath-houses bathe, one may any day see half a dozen lying in the pool side by side, one with his body covered with the most foul and loathsome ulcers, and next to him one with his skin as clear and clean as an infant's, and yet all alike seem happy, and upon the most careful inquiry I could not learn that a suspicion existed that any one had ever received injury by reason of such proximity.

It is generally claimed that the waters contain electricity. Some persons informed me that they experienced a feeling akin to a mild shock on getting into the bath, and I was informed it had been proven to exist by scientific tests, but I did not feel entirely satisfied on this point. I have a vague notion that heat and electricity are in some way correlated, but speculation upon this subject leads me away from my subject. The water as it emerges from the ground seems to possess some quality which is lost when conveyed to any considerable distance. There is an appreciable difference in the effect of a bath taken at the same temperature in a house directly over the spring supplying it as for the instance "Big Iron Bath House," and one taken in a house supplied with water conveyed through a tube for some distance. A bath at a given temperature in the house named produces a more powerful impression upon the system than at any other place tried by me. This building is a poor place to cool off in after the bath. It seems to me there should be for use at this season of the year a large, airy, well ventilated room attached to that house, but not encased in iron, where the bathers may loiter for a while before venturing into the open air. Those bathing at the holes before alluded to, solace themselves with the idea that they receive greater benefit from the baths they obtain without money and without price than do those who are fortune's favorites and pay their money for an inferior article. There is a species of sacred attachment manifested for the old "Rahl Hole," so called, and if I were the government agent having the direction of the matter it should never be disturbed, but should always remain free to all, as at present, even though it might interfere with some architectural plans. Any general impression standing the test of time, is reasonably certain to have some foundation in fact. There are some peculiarities of the water which I will not attempt to explain, but will mention one or two. Taken at a temperature of 160° and placed over a fire by the side of cold water, the last will come to a boil first. Clothing left soaking in the hot water over night, becomes so offensive as to require boiling before it can be worn.

This is not the case when soaked in the same water after it has become cold.

Hot Springs is said to have between sixty and seventy physicians. Among them are many gentlemen who would take high rank in any city of the Union, and in whose hands the profession may feel their patrons are safe in every respect. It would not be proper for me to mention names, but it is safe to advise the avoidance of those who employ extraordinary means to secure patronage. Unless those who consult you, know to whom they wish to go, or, you know some one to whom to send them, I would say advise them not to consult any one until forty-eight hours after their arrival upon the ground, and not until they have had an opportunity to obtain disinterested information. It is proverbial that invalids are easily duped in making the selection of their medical adviser. The glare of great pretensions often blinds them. I presume I do no one injustice when I say that at every place of invalid resort there are to be found so-called doctors whose highest purpose seems to be to make all the money they can out of the victims who fall into their hands, and who make a pretense of knowledge they do not possess, hence these words of caution. With these thoughts I am

Respectfully yours, J. L. WHITE.

